# SOCIOECONOMIC DISPARITIES IN PHYSICAL AND OCCUPATIONAL THERAPY HOME HEALTH CARE VISITS FOR PEOPLE WITH DEMENTIA

**DOI:** 10.1093/geroni/igad104.2355

**Published:** 2023-12-21

**Authors:** Ashwath Muruganand, Rebecca Lassell, Shih-Yin Lin, Kimberly Convery, Jason Fletcher, Abraham Brody

**Affiliations:** New York University Grossman School of Medicine, New York City, New York, United States; New York University, New York City, New York, United States; NYU Rory Meyers College of Nursing, New York City, New York, United States; NYU Rory Meyers College of Nursing, New York City, New York, United States; NYU Rory Meyers College of Nursing, New York City, New York, United States; NYU Rory Meyers College of Nursing, New York City, New York, United States

## Abstract

People living with Dementia (PLWD) utilize Home Health Care (HHC) services such as Physical Therapy (PT) and Occupational Therapy (OT), but little is known about service utilization patterns among PLWD from different socioeconomic backgrounds. We aimed to identify disparities in service utilization in PLWD in a HHC setting across socioeconomic backgrounds using a measure of geographic socioeconomic deprivation, the Area Deprivation Index (ADI). ADI is a composite measure of census variables describing socioeconomic disadvantage, reported as national percentile rank. This secondary analysis utilized data collected February 2017–May 2022 in a dementia symptom management clinical trial at a large, urban, Medicare certified nonprofit HHC agency in New Jersey. Demographic and service utilization data from 153 dyads of PLWD and caregivers was collected. PLWD’s area codes were used to calculate ADI. ADI national percentile was divided into quartiles, and PT and OT utilization for the highest quartile was compared to the three lowest quartiles using Poisson regression, consistent with previous literature. The highest quartile group was defined as ADI above the 36.5th percentile. ADI varied from 2nd to 73rd percentile, with a median of 24th percentile. Analysis controlled for pre-existing comorbidities using pre-trial Charlson Comorbidity Index scores, classified as high (>5), medium (3-4), or low (1-2). High ADI classification was associated with a 50.2% decrease in OT utilization (IRR=.498, CI=.348-.713), and 39.4% decrease in PT utilization (IRR=.606, CI=.485-.759). Further investigation of disparities in PT and OT utilization in HHC is needed, given the disproportionate burden of disease on socioeconomically distinct populations.

